# Chronic thromboembolic pulmonary hypertension – still evolving

**DOI:** 10.21542/gcsp.2020.11

**Published:** 2020-04-30

**Authors:** Mario Gerges, Magdi Yacoub

**Affiliations:** 1 Department of Internal Medicine II, Division of Cardiology, General Hospital Vienna, Medical University of Vienna, Vienna, Austria; 2 Aswan Heart Center, Aswan Governate, Egypt; 3 Department of Cardiac Surgery and Heart Science Centre, National Heart and Lung Institute, Royal Brompton and Imperial College London, London, United Kingdom

## Abstract

Chronic thromboembolic pulmonary hypertension (CTEPH) is one of the leading causes of severe pulmonary hypertension (PH). The disease is still underdiagnosed, and the true prevalence is unknown. CTEPH is characterized by intraluminal non-resolving thrombus organization and fibrous stenosis, or complete obliteration of pulmonary arteries, promoted by progressive remodeling of the pulmonary vasculature. One consequence of this is an increase in pulmonary vascular resistance and pressure, resulting in PH and progressive right heart failure, leading to death if left untreated.

Endovascular disobliteration by pulmonary endarterectomy (PEA) is the preferred treatment for CTEPH patients. PEA surgery is the only technique that can potentially cure CTEPH disease, especially in patients with fresh or organized thrombi of the proximal branches of pulmonary arteries. However, not all patients are eligible for PEA surgery. Recent research has provided evidence suggesting balloon pulmonary angioplasty (BPA) and targeted medical therapy as additional promising available treatments options for inoperable CTEPH and recurrent/persistent PH after PEA surgery.

Studies on BPA have shown it to improve pulmonary hemodynamics, symptoms, exercise capacity and RV function in inoperable CTEPH. Subsequently, BPA has developed into an essential component of the modern era of CTEPH treatment. Large randomized controlled trials have demonstrated varying significant improvements with targeted medical therapy in technically inoperable CTEPH patients. Thus, treatment of CTEPH requires a comprehensive multidisciplinary assessment, including an experienced PEA surgeon, PH specialist, BPA interventionist and CTEPH-trained radiologist at expert centers. In this comprehensive review, we address the latest developments in the fast-evolving field of CTEPH. These include advancements in imaging modalities and developments in operative and interventional techniques, which have widened the range of patients who may benefit from these procedures. The efficacy and safety of targeted medical therapies in CTEPH patients are also discussed. As the treatment options for CTEPH improve, hybrid management involving multiple treatments in the same patient may become a viable option in the near future.

## Introduction

Chronic thromboembolic pulmonary hypertension (CTEPH) is a rapidly evolving research field. Current 2015 European Society of Cardiology (ESC)/European Respiratory Society (ERS) guidelines for the diagnosis and treatment of pulmonary hypertension (PH)^1,2^, as well as the most recent proceedings of 6^th^ World Symposium on PH held in Nice, France, in 2018^3,4^, and the 2019 ESC/ERS guidelines for the diagnosis and management of acute pulmonary embolism^5,6^ have adopted new insights into the understanding of CTEPH.

Recently at the 6^th^ World Symposium, an updated hemodynamic and clinical classification of PH was presented. This most recent comprehensive clinical classification continues to divide PH into five, etiologically-oriented clinical conditions associated with PH based on similar pathophysiological mechanisms, clinical presentation, haemodynamic characteristics and therapeutic management. CTEPH is classified within group 4 of the clinical classification of PH^3,4^. Here we review the present literature on the updated hemodynamic and clinical classification of PH, epidemiology, pathophysiology, natural history, clinical presentation and diagnosis along with new treatment concepts and strategies of CTEPH.

## Hemodynamic and clinical classification

According to the current 2015 European Society of Cardiology (ESC)/European Respiratory Society (ERS) guidelines, pulmonary hypertension (PH) is a pathophysiological condition defined as an increase in mean pulmonary arterial pressure (mPAP) ≥25 mmHg, determined by resting supine right heart catheterization (RHC) at rest^1,2^.

While PH can be the primary disease itself, it mostly can be found in different conditions as comorbidity. The physiologic mPAP at rest is considered to be 14 ± three mmHg, with an upper limit of approximately 20 mmHg^1,2,7,8^. Accumulating evidence from studies on pulmonary arterial hypertension associated with systemic sclerosis showed that patients with mPAP of between 21 and 24 mmHg have symptoms comparable to those who fulfill the classic definition. These individuals have been identified to have an increased risk to progress to ≥25 mmHg and have a higher mortality than patients with mPAP <20 mmHg^9–12^.

In patients with chronic lung diseases, mildly elevated PH pressures are associated with an increased risk of death^13,14^. Moreover, comparable observations have been made in mixed patient populations^15^. The largest reported series^16^, analyzing data of more than 21,000 patients from the US veterans system, found that the hazard ratio of death for patients with mPAP between 19 and 24 mmHg compared to those with a mPAP <19 mmHg was 1.23 (95% CI [1.12–1.36]; p < 0.001). Subsequently during the 6th World Symposium on Pulmonary Hypertension, a revision of the haemodynamic definition of PH was proposed, which lowered the threshold from ≥25 mmHg to >20 mmHg (Table 1)^3^.

**Table 1:** Haemodynamic definitions of pulmonary hypertension (PH). Adapted from Simonneau et al. Haemodynamic definitions and updated clinical classification of pulmonary hypertension, Number 4 in the series “Proceedings of the 6th World Symposium on Pulmonary Hypertension” Edited by N. Galiè, V.V. McLaughlin, L.J. Rubin and G. Simonneau^3^.

Definitions	Characteristics	Clinical groups #
Pre-capillary PH	mPAP >20 mmHg PAWP ≤15 mmHg PVR ≥3 WU	1, 3, 4 and 5
Isolated post-capillary PH (Ipc-PH)	mPAP >20 mmHg PAWP ≥15 mmHg PVR <3 WU	2 and 5
Combined post-capillary PH (Cpc-PH)	mPAP >20 mmHg PAWP ≥15 mmHg PVR ≥3 WU	2 and 5

**Notes.**

mPAP mean pulmonary arterial pressure PAWP pulmonary arterial wedge pressure PVR pulmonary vascular resistance WU Wood Units

#: group 1 = PAH; group 2 = PH due to left heart disease; group 3 = PH due to lung diseases and/or hypoxia; group 4 = PH due to pulmonary artery obstructions; group 5 = PH with unclear and/or multifactorial mechanisms.

The 2^nd^ World Symposium on PH held in Evian in 1998, established a clinical classification of PH^17^. Multiple clinical conditions are categorized into five groups according to their haemodynamic characteristics, pathological findings, clinical presentation and treatment strategy^1,2,17^. During the subsequent world meetings, a series of modifications were carried out, reflecting progress in understanding of the disease^18^.

At the 5^th^ World Symposiumon PH, the general scheme of previous clinical classifications was maintained^19^. The updated clinical classification of PH presented at this Symposium was then adapted to the current 2015 ESC/ERS guidelines^1,2^. Since then, major progress has occurred in the understanding and management of chronic thromboembolic pulmonary hypertension (CTEPH). At the 6^th^ World Symposium on PH, an updated clinical classification of PH was presented. This most recent comprehensive clinical classification continues to divide PH into five, etiologically oriented clinical conditions associated with PH based on similar pathophysiological mechanisms, clinical presentation, haemodynamic characteristics and therapeutic management. CTEPH is classified within group 4 of the clinical classification of PH (Table 2 and Table 3)^3^.

**Table 2:** Updated clinical classification of pulmonary hypertension (PH). Adapted from Simonneau et al. Haemodynamic definitions and updated clinical classification of pulmonary hypertension, Number 4 in the series “Proceedings of the 6th World Symposium on Pulmonary Hypertension” Edited by N. Galiè, V.V. McLaughlin, L.J. Rubin and G. Simonneau^3^.

**1 PAH**
1.1 Idiopathic PAH
1.2 Heritable PAH
1.3 Drug- and toxin-induced PAH
1.4 PAH associated with:
1.4.1 Connective tissue disease
1.4.2 HIV infection
1.4.3 Portal hypertension
1.4.4 Congenital heart disease
1.4.5 Schistosomiasis
1.5 PAH long-term responders to calcium channel blockers
1.6 PAH with overt features of venous/capillaries (PVOD/PCH) involvement
1.7 Persistent PH of the newborn syndrome
**2 PH due to left heart disease**
2.1 PH due to heart failure with preserved LVEF
2.2 PH due to heart failure with reduced LVEF
2.3 Valvular heart disease
2.4 Congenital/acquired cardiovascular conditions leading to post-capillary PH
**3 PH due to lung diseases and/or hypoxia**
3.1 Obstructive lung disease
3.2 Restrictive lung disease
3.3 Other lung disease with mixed restrictive/obstructive pattern
3.4 Hypoxia without lung disease
3.5 Developmental lung disorders
**4 PH due to pulmonary artery obstructions (Table 3)**
4.1 Chronic thromboembolic PH
4.2 Other pulmonary artery obstructions
**5 PH with unclear and/or multifactorial mechanisms**
5.1 Haematological disorders
5.2 Systemic and metabolic disorders
5.3 Others
5.4 Complex congenital heart disease

**Notes.**

PAH pulmonary arterial hypertension PVOD pulmonary veno-occlusive disease PCH pulmonary capillary hemangiomatosis LVEF left ventricular ejection fraction

**Table 3:** Pulmonary hypertension (PH) due to pulmonary artery obstructions. Adapted from Simonneau et al. Haemodynamic definitions and updated clinical classification of pulmonary hypertension, Number 4 in the series “Proceedings of the 6th World Symposium on Pulmonary Hypertension” Edited by N. Galiè, V.V. McLaughlin, L.J. Rubin and G. Simonneau^3^.

**4.1 Chronic thromboembolic PH**
**4.2 Other pulmonary artery obstructions**
4.2.1 Sarcoma (high or intermediate grade) or angiosarcoma
4.2.2 Other malignant tumors
Renal carcinoma
Uterine carcinoma
Germ cell tumors of the testis
Other tumors
4.2.3 Non-malignant tumors
Uterine leiomyoma
4.2.4 Arteritis without connective tissue disease
4.2.5 Congenital pulmonary artery stenoses
4.2.6 Parasites
Hydatidosis

## Epidemiology

Determining the precise incidence of CTPEH is difficult. Precise prevalence and annual incidence of CTEPH are still unknown. Current guidelines on PH suggest an annual incidence of 5 cases per million people^1,2^. It is likely that CTEPH may be both underdiagnosed and the incidence of CTEPH after acute pulmonary embolism (PE) subject to overestimation, making the actual incidence difficult to quantify. Unspecific symptoms, variable numbers of preceding acute PE and the know-how required to read computed tomography pulmonary angiography (CTPA) lead to underdiagnosis^20,21^. Hence, the infrequent use of lung ventilation/perfusion lung scintigraphy (V/Q scan) leads to a further increase of underdiagnosed CTEPH cases^22,23^.

The most recent 2019 ESC/ERS guidelines for the diagnosis and management of acute PE indicate that during the first two years after a symptomatic PE episode CTEPH has a cumulative incidence of 0.1–9.1%^1, 2, 5, 6, 24–32^. In France almost 30,000 acute PE cases are diagnosed annually, with an estimated CTEPH incidence rate of 3.4%^33^. A more recent study suggested a CTEPH incidence rate of 4.8% in France^34^.

Other sources like the “Spanish PH Registry” report a prevalence and incidence rate of 3.2 cases per million and 0.9 cases per million per year, respectively^35^. A population-based cohort study in Ontario, Canada included 50,529 patients with PH from 1993 to 2012 reported a prevalence of 12.1 cases per 100,000 people^36^.

A prospective, multicentre, registry in Germany enrolled patients with newly diagnosed CTEPH within 2016. The incidence of CTEPH in Germany 2016 was then estimated at 5.7 per million of the adult population^37^.

Another prospective, multicentre, observational screening survey in Switzerland, for the detection of CTEPH included patients with acute PE from 2009 to 2016. In this study CTEPH incidence following PE was 3.7 per 1,000 patient-years, with a 2 year cumulative incidence of 0.79%^38^. The potential risk of error is mostly due to referral bias, lack of early signs and symptoms, and challenges in the differentiation of acute PE symptoms and pre-existing CTEPH^39,40^.

## Pathophysiology and natural history

CTEPH is a life-threatening disease characterized by organized non-resolving thrombotic material and progressive remodeling of the pulmonary vasculature. Altered vascular remodeling is initiated or potentiated by a combination of defective angiogenesis, impaired fibrinolysis, and endothelial dysfunction^41–43^. Incomplete thrombus resolution and organization or repeated PE seem to be a common cause^44^. It appears that unresolved emboli transform into fibrotic scar tissue and increase the vascular resistance that leads to PH^44^. Nevertheless, the exact pathogenesis of CTEPH remains unknown in most cases, but seems to be promoted by acute PE^44^.

In CTEPH, PH is due not only to the mechanical effect of proximal pulmonary artery obstruction, but also due to the remodeling of small distal arteries in the “open” pulmonary vascular bed. Lung biopsy specimens from non-occluded areas in patients with CTEPH have shown medial hypertrophy and intimal proliferation in the small distal arteries, which are the characteristic pathologic features of PAH^41^. Furthermore, patients with CTEPH have shown significant microvascular changes in 40% of cases, which is known as *secondary microvasculopathy*^45^. These changes lead to an elevation of pulmonary vascular pressure and resistance, leading to death from right heart failure if left untreated^46,47^.

**Figure 1.**
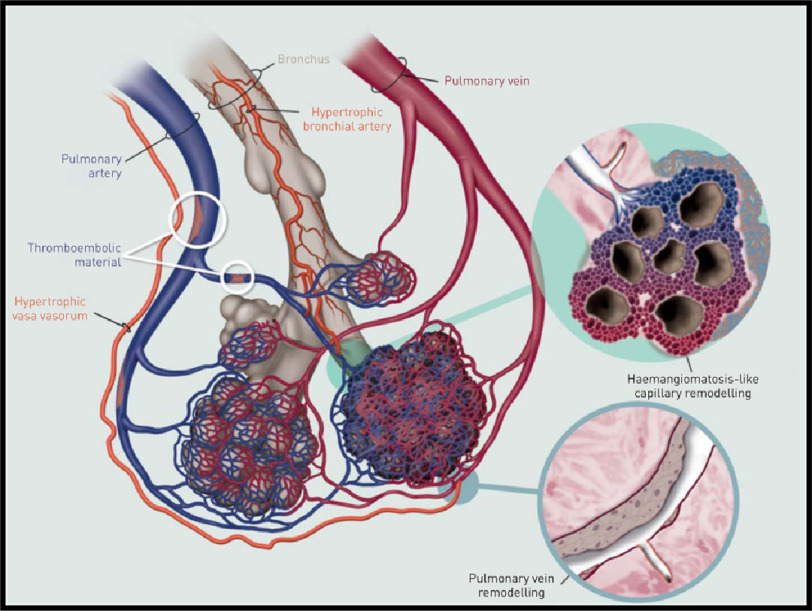
A proposed mechanism for bronchial-pulmonary venous and arterial anastomoses in chronic thromboembolic pulmonary hypertension (CTEPH). Note the schematic pre-capillary connections of systemic arteries (represented as bronchial arteries and vasa vasorum) with pulmonary arteries and with post-capillary pulmonary veins/venules. One magnified inset (top) shows the reactive thickening of the pulmonary arteriole and a hemangiomatosis-like focus featuring a multiplication of alveolar capillaries just beyond the bronchial-pulmonary arterial shunt. The other magnified inset (bottom) shows reactive fibrotic remodeling of a pulmonary vein wall at the level of a bronchial-pulmonary venous connection, relating systemic pressure with the low-pressure venous system of the lung; adapted from Dorfmüller et al. Microvascular disease in chronic thromboembolic pulmonary hypertension: a role for pulmonary veins and systemic vasculature^43^.

The development of hypertrophied bronchial arteries (BA) is a feature in CTEPH patients and might display collateral flow between the systemic and pulmonary arterial circulation^48,49^. Anastomoses are observed between bronchial arteries and pre-capillary pulmonary arterioles, post-capillary venules and small veins (Figure 1)^43^. The presence of dilated BA represents increased systemic collateral blood supply and plays an important role in maintaining the viability of ischemic lung parenchyma after pulmonary artery occlusion^48,49^.

It has been shown that total cross-sectional area of BA is associated with predominantly central CTEPH^50,51^. The collateral supply from systemic arteries downstream of pulmonary arterial occlusions contributes to microvascular remodeling, which leads to a gradual increase in PVR^43^.

However, there is no straightforward correlation between the degree of mechanical obstruction found at imaging and pulmonary hemodynamics^52^. Recent guidelines, as well as results from the international CTEPH registry, described the most frequent reported risk factors and predisposing conditions for CTEPH in the literature (Table 5)^1,2,5,6,53–55^.

**Table 4:** Chronic thromboembolic disease (CTED) compared with chronic thromboembolic pulmonary hypertension (CTEPH). Adapted from Kim et al. Chronic thromboembolic pulmonary hypertension, Number 11 in the series “Proceedings of the 6th World Symposium on Pulmonary Hypertension” Edited by N. Galiè, V.V. McLaughlin, L.J. Rubin and G. Simonneau^4^.

Diagnostic criteria	CTEPH	CTED
Symptoms	Exercise dyspnoea	Exercise dyspnoea
PH	Present at rest	Absent at rest
RHC at exercise		mPAP/CO slope >3 mmHg⋅L^−1^⋅min^−1^
V/Q scan	Any mismatched perfusion defect	Any mismatched perfusion defect
Angiography (CTPA or DSPA)	Typical findings of CTEPH	Typical findings of CTEPH
CPET		Excluding ventilatory limitation, deconditioning
TTE		Excluding left ventricular myocardial or valvular disease
Anticoagulation	At least 3 months	At least 3 months

**Notes.**

RHC right heart catheterisation V/Q scan ventilation/perfusion lung scintigraphy CTPA computed tomography pulmonary angiogram DSPA digital subtraction pulmonary angiogram CPET cardiopulmonary exercise test TTE transthoracic echocardiogram mPAP mean pulmonary arterial pressure CO cardiac output

## Clinical presentation and diagnosis

In general, pre-capillary PH is an orphan disease with high morbidity and mortality^56–59^. It is a diagnosis of exclusion, since symptoms and patient history are often unspecific^60,61^. Even though there is an increased awareness of PH, data indicate that the majority of patients are still diagnosed at a late stage of the disease^8^. A poor median survival is associated with higher WHO functional class (WHO-FC), indicating the importance of screening, correct classification and thus early diagnosis of patients with PH^57–59,62^.

A common clinical feature in early stages of CTEPH is the lack of, or unspecific nature of, symptoms. Gradually worsening shortness of breath during exertion, exercise intolerance, and general malaise has led to difficulties in diagnosis, and many patients have been misdiagnosed as having cough due to asthma bronchiale^63^.

On physical examination, findings of CTEPH are often subtle but may include a prominent pulmonary component of S2 and a systolic murmur, reflecting tricuspid regurgitation^64^. In general, transthoracic Doppler echocardiography (TTE) is the predominant screening modality in the early stages of PH diagnosis. It provides assessment of right ventricular (RV) structure and function, including the degree of ventricular remodeling as well as derivation of RV systolic and diastolic pressures and analysis of contraction timing, thus providing a reliable method for the early detection of PH^65–69^.

However, signs of right heart failure (distended neck veins, peripheral or central edema, ascites, and acrocyanosis) appear only in advanced stages of the disease^64^. Therefore, early diagnosis remains a challenge in CTEPH, with a median time of 14 months between symptom onset and diagnosis in PH expert centers^63^. If symptoms are present, clinical symptoms of CTEPH are similar to that of acute PE or pulmonary arterial hypertension (PAH) and additionally often presenting with oedema and hemoptysis. The occurrence of syncope is less frequent in CTEPH than in PAH (Table 4)^4,63^.

Often patients with chronic thromboembolic disease (CTED) show a very similar clinical picture to that of CTEPH. Patients with CTED may reveal symptoms and mismatched perfusion defects on V/Q scan, but without PH at rest. Further, exercise limitation in CTED cases has been described^70,71^.

New onset, worsened dyspnoea and persistent mismatched perfusion defects on V/Q scan often occur after acute PE in a significant proportion of patients, which makes the recognition of CTED quite challenging^72–74^. A tentative, comprehensive definition of CTED has been proposed by the 6^th^ World Symposium on PH Task Force on CTEPH (Table 4)^4^.

**Table 5:** Risk factors and predisposing conditions for chronic thromboembolic pulmonary hypertension (CTEPH). Adapted from Konstantinides et al. 2019 ESC Guidelines for the diagnosis and management of acute pulmonary embolism developed in collaboration with the European Respiratory Society (ERS), The Task Force for the diagnosis and management of acute pulmonary embolism of the European Society of Cardiology (ESC)^5,6^.

Findings related to the acute PE event (obtained at PE diagnosis)	Concomitant chronic diseases and conditions predisposing to CTEPH (documented at PE diagnosis or at 3–6 month follow-up)
Previous episodes of PE or DVT	Ventriculo-atrial shunts
Large pulmonary arterial thrombi on CTPA	Infected chronic i.v. lines or pacemakers
Echocardiographic signs of PH/RV dysfunction	History of splenectomy
CTPA findings suggestive of pre-existing chronic thromboembolic disease	Thrombophilic disorders, particularly antiphospholipid antibody syndrome and high coagulation factor VIII levels
	Non-O blood group
	Hypothyroidism treated with thyroid hormones
	History of cancer
	Myeloproliferative disorders
	Inflammatory bowel disease
	Chronic osteomyelitis

**Notes.**

CTEPH Chronic thromboembolic pulmonary hypertension CTPA computed tomographic pulmonary angiography DVT deep vein thrombosis i.v. intravenous LV left ventricular PE pulmonary embolism PH pulmonary hypertension RV right ventricular

Distinguishing between CTEPH and “subacute” PE can be challenging. Therefore, the diagnosis of CTEPH is based on findings obtained after at least 3 months of effective therapeutic anticoagulation, in order to discriminate this condition from “subacute” PE^1,2,5,6^. As already mentioned, currently a new hemodynamic threshold for PH has been proposed by the 6^th^ World Symposium on PH Task Force on PH diagnosis and classification^3^. There is sufficient evidence to update the hemodynamic threshold for PH, but the implications for CTEPH and CTED are not yet known^4^.

Mismatched perfusion defects on V/Q scan are the hallmark of CTEPH. A normal V/Q scan effectively excludes CTEPH with a sensitivity of 90–100% and a specificity of 94–100%^75,76^. Therefore, V/Q scan remains the preferred, guideline-recommended, initial imaging test for CTEPH screening^1,2,4–6^.

Other specific diagnostic signs for CTEPH are also gathered by multidetector CTPA, magnetic resonance imaging (MRI) or digital subtraction pulmonary angiography (DSPA)^1,2,4–6^. Recent data studied the diagnostic accuracy of three-dimensional dynamic contrast-enhanced lung perfusion MRI against planar V/Q scan or V/Q single-photon emission CT (SPECT) scan as a screening tool for CTEPH^77,78^. These data showed that dynamic contrast-enhanced lung perfusion MRI has a similar sensitivity (97%) for diagnosing CTEPH when compared with planar V/Q scan and a higher sensitivity (100% versus 97%) when compared with SPECT scan^77,78^. DSPA had been considered the gold standard for characterizing vessel morphology in CTEPH.

**Figure 2.**
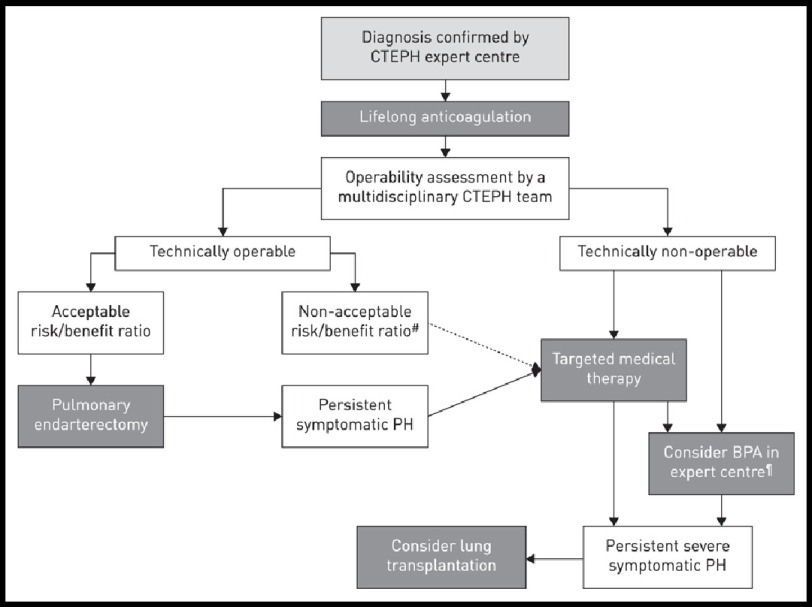
Diagnostic algorithm for chronic thromboembolic pulmonary hypertension (CTEPH). Adapted from Galie et al. 2015 ESC/ERS Guidelines for the diagnosis and treatment of pulmonary hypertension, The Joint Task Force for the Diagnosis and Treatment of Pulmonary Hypertension of the European Society of Cardiology (ESC) and the European Respiratory Society (ERS), Endorsed by: Association for European Pediatric and Congenital Cardiology (AEPC), International Society for Heart and Lung Transplantation (ISHLT)^1,2^. CT, computed tomography; CTEPH, chronic thromboembolic pulmonary hypertension; PA, pulmonary arterial hypertension; PH, pulmonary hypertension; V/Q, ventilation/perfusion. ^a^CT pulmonary angiography alone may miss diagnosis of chronic thromboembolic pulmonary hypertension.

Currently CTPA is commonly used for the assessment of operability^4^. Recent reports indicate that CTPA has a high sensitivity and specificity in detecting chronic thromboembolic lesions at the main/lobar (89–100% and 95–100%, respectively) and segmental (84–100% and 92–99%, respectively) levels^79,79–81^. The current algorithm for CTEPH diagnosis is in the 2015 ESC/ERS guidelines (Figure 2)^1,2^.

**Figure 3.**
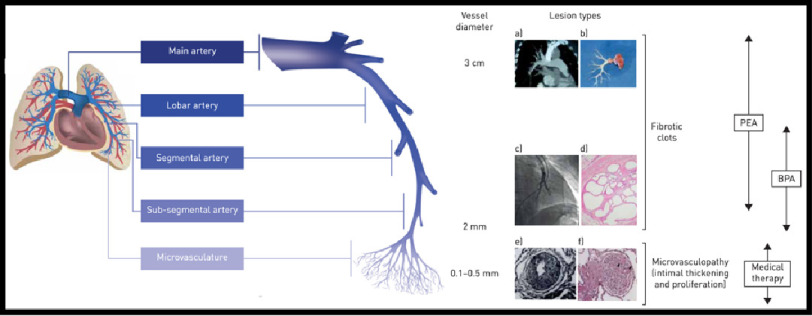
The management options for chronic thromboembolic pulmonary hypertension (CTEPH) target different pathogenic manifestations in different parts of the pulmonary vascular bed. A schematic representation of a pulmonary artery is shown (note that vessel diameter is not to scale). Pulmonary endarterectomy (PEA) is used to remove thromboembolic lesions primarily in the proximal main artery (diameter of ∼3 cm), and lobar and segmental arteries^84,140^; in expert surgical centres, lesions in distally located mid-segmental and sub-segmental branches can be targeted by PEA^84^, down to vessels of two mm in diameter. Balloon pulmonary angioplasty (BPA) mainly targets distal lesions in the segmental and sub-segmental vasculature, down to small pulmonary arteries of 2–5 mm in diameter. Medical therapy targets microvasculopathy, including intimal thickening and fibromuscular proliferation, in vessels of 0.1–0.5 mm in diameter.^44^ A) Computed tomography scan of a pulmonary artery. B) Organized fibrotic material removed during PEA. C) Selective pulmonary angiogram of segmental and sub-segmental pulmonary arteries, showing irregular vessel contour and occlusion, typical of CTEPH. D) Microscopic examination showing a luminal filling defect with recanalised chronic thrombus (web lesion) and no evidence of vasculopathy in the sub-segmental artery. E) Intimal fibromuscular proliferation.^41^ F) Plexiform lesion and vessel occlusion due to vasculopathy and proliferation; Adapted from Madani et al. The changing landscape of chronic thromboembolic pulmonary hypertension management^82^.

With respect to disease-specific management and treatment, surgical, percutaneous interventional and/or pharmacological options are currently available to target proximal lesions, distal lesions and microvasculopathy (Figure 3)^82^. Individual CTEPH patients are likely to exhibit more than one of these disease manifestations and therefore may benefit from more than one treatment approach. The currently available options for CTEPH management and treatment are the focus of the remainder of this article.

## Treatment Strategies

### Surgical treatment: pulmonary endarterectomy

Pulmonary endarterectomy (PEA), whose efficacy began to be recognized around 1980, is the only technique that can potentially cure CTEPH disease, especially in patients with fresh or organized thrombi of the proximal branches of pulmonary arteries (types 1 and 2 of the surgical classification)^83^.

Surgical treatment by PEA is the treatment of choice for CTEPH and should be offered to all suitable CTEPH patients. PEA provides the greatest impact of symptomatic and prognostic improvement in eligible CTEPH patients with excellent results in specialist centers^84^. The 6^th^ World Symposium on PH Task Force on CTEPH provided a newly proposed CTEPH treatment algorithm^4^. Compared with the surgical embolectomy for acute PE, bilateral endarterectomy through the medial layer of the pulmonary arteries is required to successfully treat CTEPH. This surgical procedure has been previously described in detail^26,84–94^.

**Figure 4.**
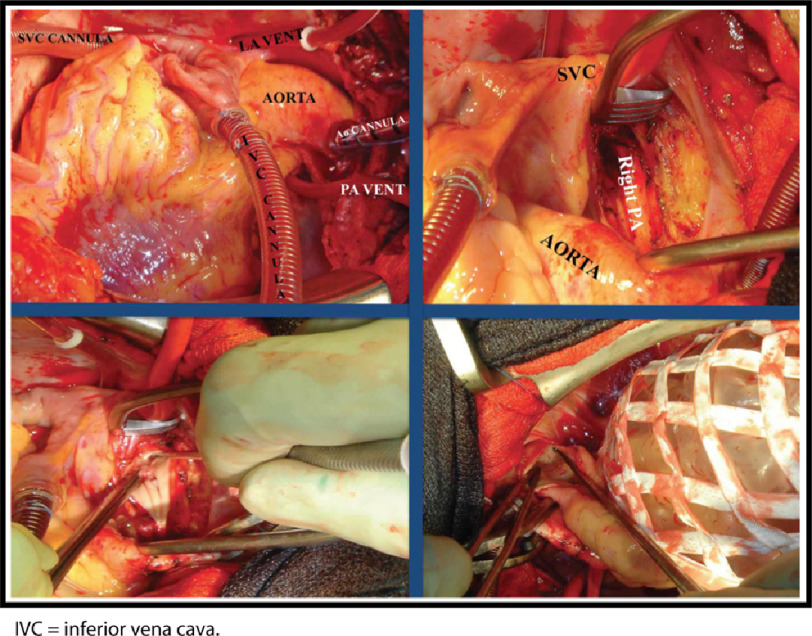
Surgical technique of pulmonary endarterectomy. (A) Intraoperative set-up for pulmonary endarterectomy. Initially, the surgeon stands on the left side of the patient. In addition to cannula in both inferior and superior vena cava (SVC), pulmonary artery (PA) and left atrial (LA) vents are also placed. The LA vent is placed via the right superior PA and directed into the left ventricle while the patient is cooling and the heart fibrillating. (B) Surgical approach to the right PA and endarterectomy. The right PA is approached between the aorta and SVC and not lateral to the SVC. This gives better exposure of the more distal branches. (C) The plane of dissection is raised posteriorly initially and then a complete endarterectomy performed. (D) Surgical approach to the left PA. The heart is retracted medially using a mesh retractor, and the left PA is exposed. The arteriotomy is then directed toward the descending PA past the upper lobe take-off. Again, the endarterectomy is initiated by raising the correct plane over the posterior aspect of the vessel; Adapted from Madani et al. Pulmonary Endarterectomy. Patient selection, technical challenges, and outcomes.^94^

**Figure 5.**
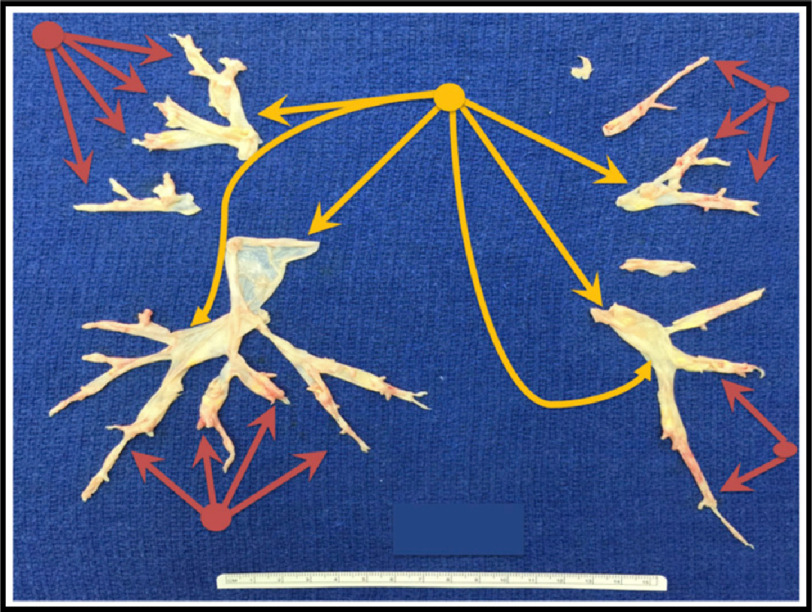
Endarterectomy specimen from a patient with subsegmental disease. Note the plane of endarterectomy starting in the areas of normal vessel wall within the main lobar branches (yellow arrows) gives the surgeon the ability to reach the distally located obstructive material hidden from the view (red arrows); Adapted from Madani et al. Pulmonary Endarterectomy. Patient selection, technical challenges, and outcomes.^94^.

**Figure 6.**
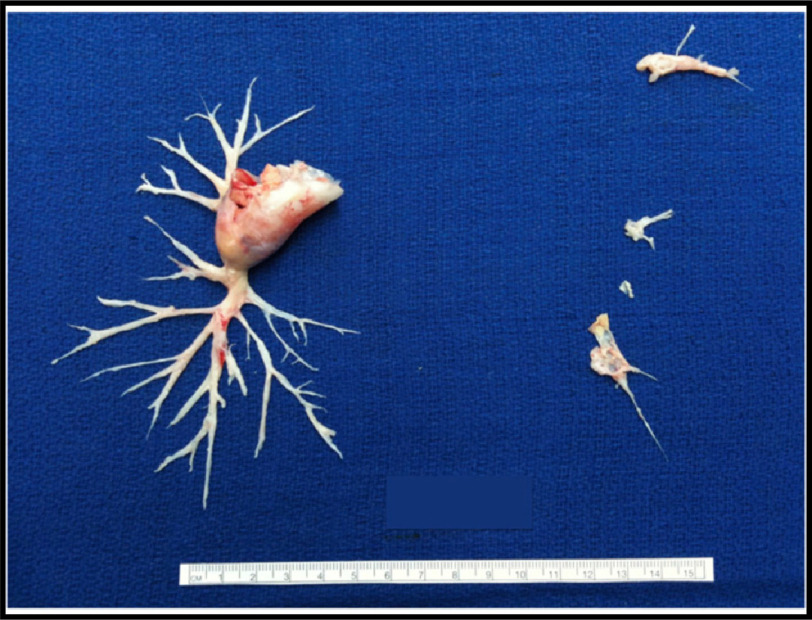
Surgical specimen removed from a patient with complete occlusion on the right side and distal obstruction on the left side. Note that removal of proximal material without a complete endarterectomy into the distal branches will not be effective. The ruler measures 15 cm. The specimen is characterized as level IC disease on the right side and level III on the left side; Adapted from Madani et al. Pulmonary Endarterectomy. Patient selection, technical challenges, and outcomes.^94^.

Briefly, after cardiopulmonary bypass is established, deep hypothermia between 18^∘^C and 20^∘^C is induced. The endarterectomy is performed during intermittent circulatory arrest, without need for cerebral perfusion, to avoid bleeding from systemic-to-pulmonary collaterals. The surgeon establishes the correct endarterectomy plane, which is followed down to lobar, segmental, or subsegmental branches of each lobe (Figures 4, 5 and 6^26,84–94^.

Current surgical advances led to an in-hospital mortality rate of <4.7%^95^ and even lower in high-volume centers^96^. A substantial relief from symptoms and near-normalization of hemodynamics can be achieved in the majority of surgically-treated patients^84,95–97^.

PEA is carried out in expert centers due to the complexity of the surgical procedure and peri-procedural patient management. Eligibility for surgery includes a decision made at a dedicated multidisciplinary CTEPH team meeting including an experienced PEA surgeon, PH specialist, BPA interventionist and CTEPH-trained radiologist^4^. The operability of CTEPH patients is determined by many variables that cannot be standardized easily. Such considerations are related to the patient’s suitability, the surgical team’s experience and available resources. General criteria include pre-operative World Health Organization (WHO) functional class II-IV and the surgical accessibility of thrombi in the main, lobar, or segmental pulmonary arteries^95^. Advanced age alone is not a contraindication for surgery and there is no PVR threshold or measure of RV dysfunction that can be considered to preclude PEA^1,2,5,6^.

The use of post-operative ECMO is recommended as current standard of care in PEA centers for severe cases^83,84,98,99^. Early post-operative reperfusion edema after PEA surgery may require veno-arterial ECMO^1,2,5,6^. Moreover, severe persistent PH may be bridged with ECMO to emergency lung transplantation. It is recommended that patients after performed PEA surgery should be followed in CTEPH centers to exclude persistent or recurrent PH, with at least one haemodynamic assessment to be considered at 6-12 months after the intervention^1,2,5,6^.

Recently, the international registry reported survival rates in incident CTEPH patients. A 3-year survival rate has been reported for 90% of all operated CTEPH patients and in 70% in those who did not receive surgical treatment^100^. Data from a large cohort reported a long-term follow-up of 10-year survival of 72%^101^. Further, death was attributed to unrelated causes in 49% of patients and residual PH with PVR ≥425 dyn⋅s⋅cm^−5^ correlated with worse outcome^101^.

From the current standpoint, strict objective definitions of operability remain impossible, however certain features are more likely to predict a good surgical outcome (Table 6)^4^. Patients may be technically operable, but they might not benefit from PEA due to significant comorbidities. Until now the best treatment option for inoperable CTEPH patients with a non-acceptable risk/benefit ratio remain unclear^4^. Inferior vena cava filter (IVC) device insertion prior to PEA has not been properly studied. Therefore, this routine practice of IVC device implantation has been abandoned at the leading centers. Data from the international CTEPH registry revealed that IVC filter prior to PEA surgery did not impact on long-term survival^101^.

**Table 6:** Favorable risk–benefit assessment for pulmonary endarterectomy (PEA). Adapted from Kim et al. Chronic thromboembolic pulmonary hypertension, Number 11 in the series “Proceedings of the 6th World Symposium on Pulmonary Hypertension” Edited by N. Galiè, V.V. McLaughlin, L.J. Rubin and G. Simonneau^4^.

Characteristics	Lower risk with predictable good long-term outcome	Higher risk with less predictable long-term outcome (not contraindications)
History	History of DVT/PE	No history of DVT/PE
Examination	No signs of right heart failure	Signs of right heart failure
Comorbidity	None	Significant concomitant lung or left heart disease
Functional limitation	Functional class II or III	Functional class IV
Imaging	Clear disease concordant on all images	Inconsistency on imaging modalities
Type of disease	Bilateral lower lobe disease	No disease appreciable in lower lobes
Hemodynamics	PVR <1000 dyn⋅s⋅cm^−5^, in proportion to site and number of obstructions on imaging; higher PA pulse pressure	PVR >1200 dyn⋅s⋅cm^−5^, out of proportion to site and number of obstructions on imaging; higher PA diastolic pressure

**Notes.**

DVT deep vein thrombosis PE pulmonary embolism PVR pulmonary vascular resistance PA pulmonary artery

**Figure 7.**
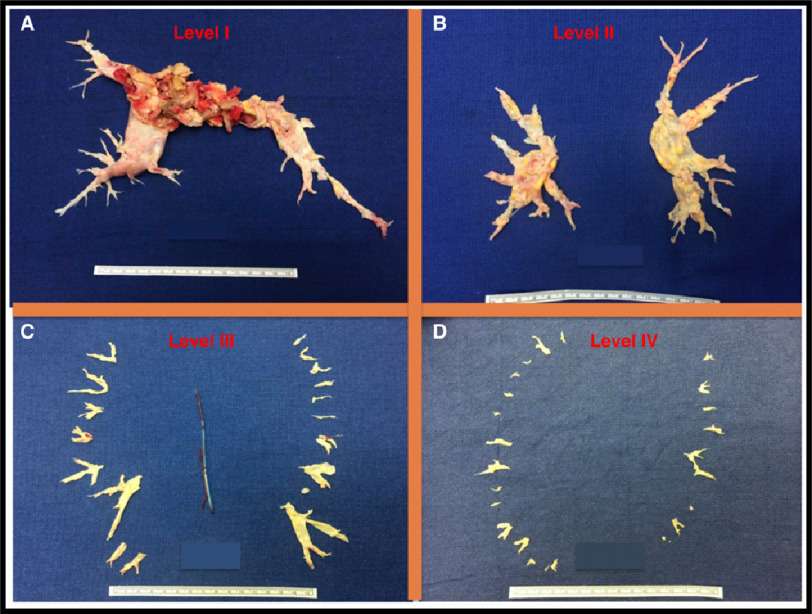
University of California, San Diego classification of pulmonary endarterectomy disease levels. This figure illustrates typical surgical specimens classified based on the most proximal level of obstruction, levels I to IV; Adapted from Madani et al. Pulmonary Endarterectomy. Patient selection, technical challenges, and outcomes.^94^.

Over the past decades important surgical advances have redefined the distal limits of endarterectomy^93,94^. Based on the evolving surgical techniques in expert facilities, patients with distal chronic thromboembolism have been successfully treated^93^. Advances in invasive and non-invasive diagnostics and increasing surgical expertise have added further to this progress. Hence, the previously published intra-operative classification^83^ has been refined by the by the 6^th^ World Symposium on PH Task Force on CTEPH to better reflect the ability of the current surgical approach and level of revascularization (Table 7 and Figures 6 and 7)^4,94^.

**Table 7:** University of California San Diego chronic thromboembolism (CTE) surgical classification. Adapted from Madani et al. Pulmonary endarterectomy. Patient selection, technical challenges, and outcomes.^94^, and Kim et al. Chronic thromboembolic pulmonary hypertension, Number 11 in the series “Proceedings of the 6th World Symposium on Pulmonary Hypertension” Edited by N. Galiè, V.V. McLaughlin, L.J. Rubin and G. Simonneau^4^.

Surgical levels	Location of CTE
Level 0	No evidence of thromboembolic disease in either lung
Level I	CTE starting in the main pulmonary arteries
(Level IC)	(Complete occlusion of one main pulmonary artery with CTE)
Level II	CTE starting at the level of lobar arteries or in the main descending pulmonary arteries
Level III	CTE starting at the level of the segmental arteries
Level IV	CTE starting at the level of the subsegmental arteries

Consequently, not all surgical centers will view operability in the same manner because treatment of patients with CTEPH depends largely on subjective judgments of eligibility for surgery by the CTEPH team^84^. The 6^th^ World Symposium on PH Task Force on CTEPH proposed a three-step stratified definition of an expert surgical center by the following important goals: surgical mortality (< 5%), surgical volume (more than 50 PEAs per year) and ability to perform segmental endarterectomy^84^. Furthermore, an expert center should be capable of evaluating and offering any/all established treatment modalities according to individual need^4^.

The role of PH-targeted pharmacological treatment and BPA relative to PEA surgery is not clearly established and relies on the anatomical distribution of pathology. The combination of both techniques PEA and BPA either as a composite or stepwise approach is currently being evaluated at select expert programmes^102^. Regarding PH-targeted medical therapy, the CHEST-1 study showed that riociguat was beneficial for patients with residual PH after PEA^103^. Currently an ongoing randomised, double-blind, placebo-controlled, multicentre, multinational, prospective study is evaluating PH-targeted medical therapy prior to PEA in operable patients (ClinicalTrials.gov Identifier: NCT03273257).

### Interventional treatment: balloon pulmonary angioplasty

Balloon pulmonary angioplasty (BPA) was first developed for treating congenital pulmonary artery (PA) stenosis^104^. It was attempted in a case series of seven children with either PA stenosis or hypoplasia with reported improvements in RV pressure, decrease in intravascular gradient across the obstruction and increase in diameter of the narrowed PA segment. An increase of the percentage blood flow to the dilated lung region, as measured by quantitative V/Q scan, was also described^104^.

After that the first BPA procedure in CTEPH was reported in Europe 1988^105^, and in then in the U.S. in 2001^106^, but was abandoned early because of the high rate of complications. The initial case report from 1988 demonstrated a 30-year-old man with inoperable CTEPH treated with BPA^105^. Mean PAP could be reduced from 46 mmHg to 35 mmHg with concomitant increase of mean aortic pressure from 75 mmHg to 90 mmHg^105^. Two more BPA procedures were performed followed by transient segmental pulmonary edema^105^.

More than 10 years later, in 2001, results were reported in a case series of 18 inoperable CTEPH patients^106^. After an average of 36 months of follow-up, the average New York Heart Association (NYHA) class improved from 3.3 to 1.8, 6-minute walking distances (6MWD) increased from 209 to 497 yards and the mPAP improved from 43.0 ± 12.1 mmHg to 33.7 ± 10.2 mmHg, all statistically significant.

Although efficacy of BPA could be demonstrated, the complication rate was high at 61%. 11 patients developed pulmonary edema, 3 patients needed mechanical ventilation management and 1 died due to RV failure^106^.

In the meantime, Japanese colleagues launched a study in 2004 which reported treatment results of 68 CTEPH patients in 2012^107^. Compared to the report from 2001^106^, colleagues from Japan refined the technique of BPA^107–109^. Since the Japanese researchers presented their results in 2012, BPA has developed into an essential component of the modern era of CTEPH treatment algorithm^107–109^. Subsequently, studies on BPA have shown to improve pulmonary hemodynamics, symptoms, exercise capacity and RV function (Tables 8 and 9)^107–124^. Furthermore, after refinement of BPA, significantly lower rates of major complications could be achieved^113,116,125^ compared with the report from 2001^106^.

**Table 8:** Overview of reported balloon pulmonary angioplasty (BPA) case reports, patient series and studies.

Author	Publication year	Study location	Study design	Number of patients	Diagnosis	Medical treatment before BPA
Voorburg et al.^105^	1988	The Netherlands	Case report	1	CTEPH	-
Feinstein et al.^106^	2001	United States	Observational	18	CTEPH	100%
Mizoguchi et al.^107^	2012	Japan	Observational	68	CTEPH	100%
Kataoka et al.^108^	2012	Japan	Observational	29	CTEPH	100%
Sugimura et al.^109^	2012	Japan	Observational	12	CTEPH	100%
Andreassen et al.^110^	2013	Norway	Observational	20	CTEPH	10%
Inami et al.^111^	2014	Japan	Retrospective	136	CTEPH	85%
Taniguchi et al.^112^	2014	Japan	Retrospective	29	CTEPH	100%
Fukui et al.^113^	2014	Japan	Retrospective	20	CTEPH	75%
Roik et al.^114^	2014	Poland	Case report	1	CTEPH	100%
Bouvaist et al.^115^	2014	France	Case report	1	CTEPH	100%
Fukui et al.^116^	2015	Japan	Retrospective	25	CTEPH	56%
Inami et al.^117^	2016	Japan	Retrospective	170	CTEPH	91%
Aoki et al.^118^	2017	Japan	Retrospective	77	CTEPH	96%
Ogo et al.^119^	2017	Japan	Retrospective	80	CTEPH	61%
Ogawa et al.^120^	2017	Japan	Retrospective	249	CTEPH	72%
Olsson et al.^121^	2017	Germany	Retrospective	56	CTEPH	93%
Kurzyna et al.^122^	2017	Poland	Retrospective	56	CTEPH	80%
Wiedenroth et al.^123^	2018	Germany	Prospective	35	CTED	0%
Brenot et al.^124^	2019	France	Retrospective	184	CTEPH	62%

**Table 9:** Efficacy of balloon pulmonary angioplasty (BPA).

Author	Publication year	Study location	Study design	Number of patients	PVR (dyn.s.cm^−5^) before BPA	PVR (dyn.s.cm^−5^) after BPA	Treatment effect
Voorburg et al.^105^	1988	The Netherlands	Case report	1	688	532	-23%
Feinstein et al.^106^	2001	United States	Observational	18	-	-	-
Mizoguchi et al.^107^	2012	Japan	Observational	68	942 ±367	327 ±151	-65%
Kataoka et al.^108^	2012	Japan	Observational	29	-	-	-
Sugimura et al.^109^	2012	Japan	Observational	12	971 ±500	310 ±73	-68%
Andreassen et al.^110^	2013	Norway	Observational	20	704 ±320	472 ±288	-33%
Inami et al.^111^	2014	Japan	Retrospective	136	-	-	-
Taniguchi et al.^112^	2014	Japan	Retrospective	29	763 ±308	284 ±128	-63%
Fukui et al.^113^	2014	Japan	Retrospective	20	889 ±365	490 ±201	-45%
Bouvaist et al.^115^	2014	France	Case report	1	640	320	-50%
Roik et al.^114^	2014	Poland	Case report	1	848	-	-
Fukui et al.^116^	2015	Japan	Retrospective	25	755 ±345	-	-
Inami et al.^117^	2016	Japan	Retrospective	170	-	-	-
Aoki et al.^118^	2017	Japan	Retrospective	77	800	304	-62%
Ogo et al.^119^	2017	Japan	Retrospective	80	880 ±424	408 ±184	-54%
Ogawa et al.^120^	2017	Japan	Retrospective	249	854 ±451	360 ±223	-58%
Olsson et al.^121^	2017	Germany	Retrospective	56	591 ±286	440 ±279	-26%
Kurzyna et al.^122^	2017	Poland	Retrospective	56	824 ±296	472 ±230	-43%
Wiedenroth et al.^123^	2018	Germany	Prospective	35	234 ±68	167 ±40	-29%
Brenot et al.^124^	2019	France	Retrospective	184	604 ±226	329 ±177	-43%

**Notes.**

PVR pulmonary vascular resistance

**Figure 8.**
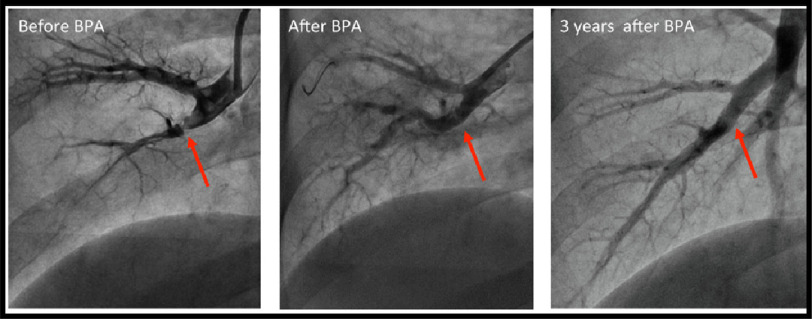
Representative selective pulmonary angiogram after balloon pulmonary angioplasty (BPA). Adapted from Matsubara et al. Balloon pulmonary angioplasty^126^.

Retrospective analyses showed the benefits of BPA seem to be maintained^117,120^. A representative selective pulmonary angiogram is shown in Figure 8 as an example of maintained results after BPA^126^. Similar findings appeared in subsequent publications from Europe^110, 114, 115, 121–124^. In Germany, significant improvements could be also shown in CTED patients^123^, but this observation needs to be further confirmed by subsequent studies on BPA in CTED. The latest experiences from Germany^121,123^ and France^124^ are remarkable, as these centers have introduced BPA along with a well-established PEA program. Although complication rates in the German series were similar to those from Japan, the magnitude of efficacy (e.g., PVR reduction) was less (Table 9). Possible reasons for these reported differences included the possibility of variations in operability level and variations in CTEPH types of patients treated with BPA between the centers^4^.

Although these BPA findings are promising, the studies come from specialist centers and cannot be generalized. Even with the technical refinements, a steep learning curve remains in order to provide BPA safely, effectively and consistently^127^. A successful BPA calls for a significant amount of training and case knowledge^4^. BPA should be exclusively performed in specialist centers where symptomatic inoperable CTEPH patients with distal recurrent thromboembolism or persistent/recurring PH are to be treated. The role of BPA has yet not been determined for those with clinically operable disease who are not eligible for surgery because of individual decision or patient refusal^4^.

A multidisciplinary CTEPH team in an expert center should review all available and applicable evidence for every BPA patient selection^4^. This includes the critical step of identifying target vessels and lesions. Therefore, the study of anatomical and functional examination of pulmonary arteries and lung perfusion are essential^128^. It is recommended to use selective pulmonary angiogram of the target vessels to visualize more details and it serves as confirmation prior to intervention during BPA procedure^4,128^. Selective angiogram may not be able to identify all distal lesions that theoretically contribute to a successful BPA. This circumstance makes it necessary to use multiple complementary imaging modalities like intravascular imaging and pressure gradient analysis to aid in lesion assessment and balloon sizing^129^.

**Figure 9.**
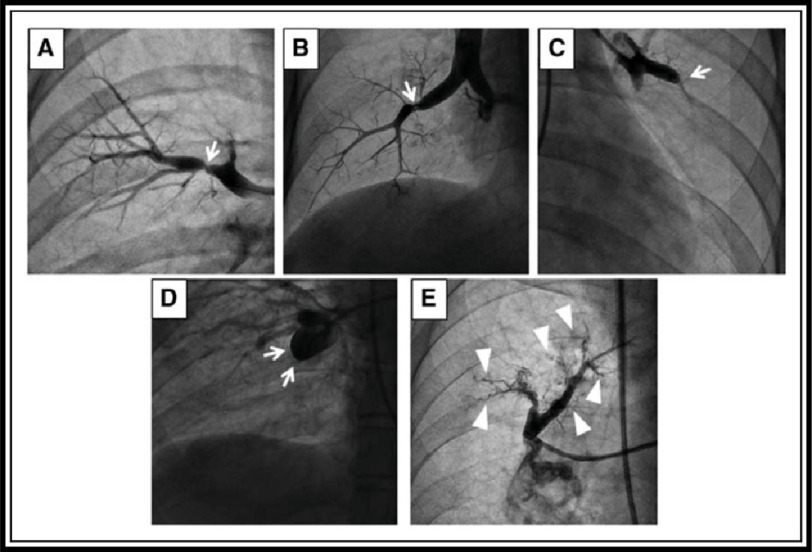
Angiographic classification of lesion morphology based on the lesion opacity and the blood flow distal to the lesion. (A) Ring-like stenosis lesion. (B) Web lesion. (C) Subtotal lesion. (D) Total occlusion lesion. (E) Tortuous lesion. Type A–D lesions are located proximal to the subsegmental pulmonary artery, namely, the segmental and subsegmental arteries. Type E lesions are located distal to the subsegmental artery; Adapted from Kawakami et al. Novel angiographic classification of each vascular lesion in chronic thromboembolic pulmonary hypertension based on selective angiogram and results of balloon pulmonary angioplasty^128^.

Japanese colleagues developed an angiographic classification of lesion morphology based on the lesion opacity and the blood flow distal to the lesion. Angiographical findings of lesions types in CTEPH are: ring-like stenosis lesions, web lesions, subtotal lesions, total occlusion lesions and tortuous lesions. In general, unsuitable lesions types for BPA are pouch lesions (Figure 9)^128^.

The 6^th^ World Symposium on PH Task Force on CTEPH calls for the definition and uniformity of BPA complication reports. A guide has recently been proposed for BPA centers for the classification of complications (Table 10)^4^. Unlike a PEA reperfusion lung injury, that may be delayed for days before onset, a BPA-related injury tends to be more vascular than the post-PEA capillary leak syndrome^130^. Wire perforation or interruption of the diseased vessel is the most common form of BPA-related injuries vascular injuries^128,131^. Experts report that lung injury through wire perforation or balloon overdilatation in the setting of severe PH risks potentially lethal massive infiltration and/or hemorrhage that may require mechanical ventilation or extracorporeal support^4^.

**Table 10:** Balloon pulmonary angioplasty (BPA) complications. Adapted from Kim et al. Chronic thromboembolic pulmonary hypertension, Number 11 in the series “Proceedings of the 6th World Symposium on Pulmonary Hypertension” Edited by N. Galiè, V.V. McLaughlin, L.J. Rubin and G. Simonneau^4^.

**During the procedure**
Vascular injury^a^ with/without hemoptysis
Wire perforation
Balloon overdilatation
High-pressure contrast injection
Vascular dissection
Allergic reaction to contrast
Adverse reaction to conscious sedation/local anesthesia
**After the procedure**
Lung injury^b^ (radiographic opacity with/without hemoptysis, with/without hypoxemia)
Renal dysfunction
Access site problems

**Notes.**

a Signs of vascular injury: extravasation of contrast, hypoxaemia, cough, tachycardia, increased pulmonary arterial pressure.

b Causes of lung injury: vascular injury much greater than reperfusion lung injury.

Reperfusion lung injury is rare with refined BPA technique. The low BPA complication rate published so far, reflects limited experience from experienced BPA centres. Thus, in experienced hands, BPA has now become a promising and established treatment option for inoperable CTEPH^4^.

### Medical treatment

For most CTEPH patients, surgical PEA remains the treatment of choice. However, numbers from the international CTEPH registry indicate that around 40% of the patients have been classified as inoperable^54^. Reasons given for inoperability include inaccessible vascular obstruction, PAP out of proportion to morphological lesions, and significant prohibitive comorbidities^54^.

Small^132^ and large randomized controlled trials have demonstrated varying significant improvements with targeted medical therapy in technically inoperable CTEPH patients^103, 133–135^. Despite that, evidence is scarce for patients with medical contraindications or those refusing surgery^4^.

Based on the CHEST studies, riociguat is the currently approved pharmaceutical therapy for inoperable CTEPH in many countries^103,136^. The MERIT-1 trial of macitentan in the treatment of inoperable CTEPH demonstrated significant improvements of the primary and other end-points as well as N-terminal pro-brain natriuretic peptide^134^. The MERIT-1 trial anticipated the first evidence on combination medical therapy in CTEPH. At the time of inclusion 61% of patients were already treated with phosphodiesterase type 5 inhibitors and/or oral/inhaled prostanoids. A further addition of macitentan demonstrated comparable results compared with the drug-naive treatment arm^134^. Hence, macitentan is now being considered for future CTEPH registration. Until now, event-driven morbidity/mortality trials have not been performed in CTEPH^4^.

In the BENEFIT and CHEST-1 trial, patients with persistent/residual PH after PEA surgery were also included; representing about 30% of the study population^103,133^. Both trials included patients >6 months after PEA surgery with mPAP ≥25 mmHg and PVR ≥ 300 dyn⋅s⋅cm^−5^ ^103,133^. Results from the BENEFIT and CHEST-1 trial can be seen with real-life data from the large UK national cohort. The UK database showed that 51% of the patients had mPAP ≥ 25 mmHg 3–6 months after PEA. Further, mPAP ≥30 mmHg predicted initiation of PH-targeted medical therapy, and mPAP ≥38 mmHg and PVR ≥425 dyn⋅s⋅cm^−5^ correlated with worse long-term survival^101^.

The use of medical therapy as a “bridge to PEA” is more a topic of controversies, because it seems that it delays timely surgical referral and, therefore, definitive optimal treatment^4^. Patients in the international CTEPH were in 28% on PH-targeted medication(s) at the time of PEA referral^54^. Similarly, the University of California San Diego cohort reported 37%, of patients were on some form of PH-targeted drug(s) at the time of surgical referral^137^. For both cohorts, the interval from diagnosis to surgery in pre-treated patients has doubled without any demonstrable clinical benefit. Moreover, in the international CTEPH registry, pre-treatment independently predicted worse outcome^100^.

The key drawbacks of these studies are their inherent reference bias and the possibility of stabilizing deteriorating cases by using medical treatment^4^. Therefore, a phase 2 trial will be soon launched to include CTEPH patients with high PVR for pre-operative care with riociguat versus placebo in order to provide the missing proof of evidence (ClinicalTrials.gov identifier NCT0327357)^4^.

While not studied yet, the use of PH-targeted therapy as ”bridge to BPA” has become common practice following the current recommendation for riociguat for technically inoperative CTEPH patients^4^. To close this gap, a study is currently underway evaluating riociguat versus BPA for technically inoperable CTEPH, followed by an opportunity to crossover after 6 months (ClinicalTrials.gov identifier NCT02634203)^4^.

### Newly-propsed treatment algorithm

**Figure 10.**
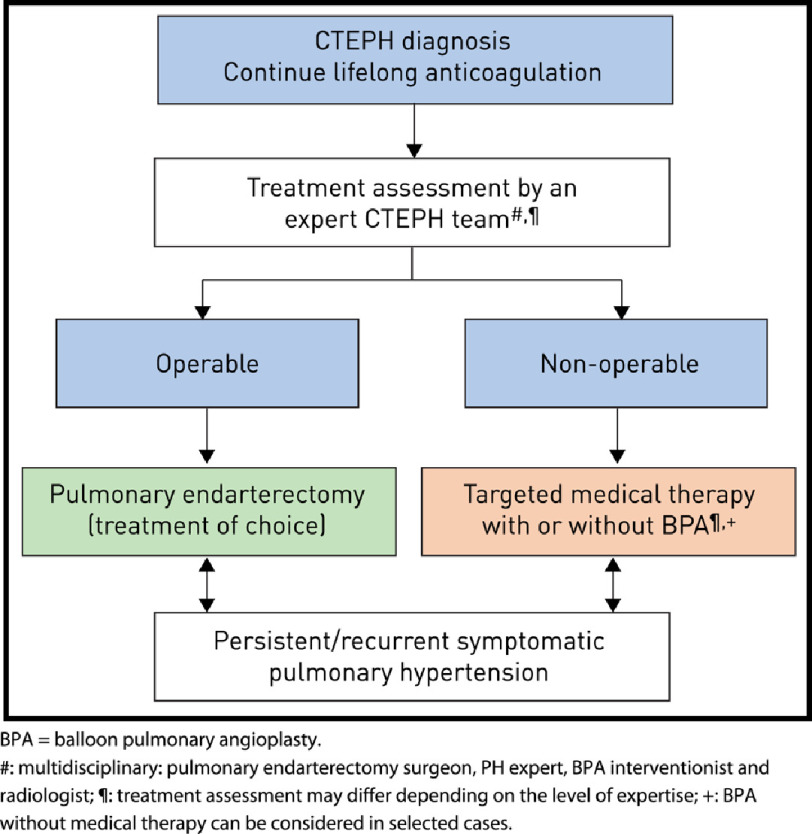
Chronic thromboembolic pulmonary hypertension (CTEPH): revised treatment algorithm. Adapted from Kim et al. Chronic thromboembolic pulmonary hypertension, Number 11 in the series “Proceedings of the 6th World Symposium on Pulmonary Hypertension” Edited by N. Galiè, V.V. McLaughlin, L.J. Rubin and G. Simonneau^4^.

The 6th World Symposium on PH Task Force on CTEPH proposed a new CTEPH treatment algorithm (Figure 10)^4^. The newly addressed treatment algorithm for CTEPH starts with lifelong anticoagulation with oral vitamin K antagonists^4^. Antiplatelet therapy in patients with CTEPH is not an alternative to traditional anticoagulation. Until now, data on the best form of anticoagulation therapy have not been presented. In CTEPH, it is unclear if new oral anticoagulants, or chronic injectable anticoagulant agents are suitable^4^. The newly proposed treatment algorithm emphasizes the need for a multidisciplinary assessment, including an experienced PEA surgeon, PH specialist, BPA interventionist and CTEPH-trained radiologist^4^. Previously, a PH referral center was defined and recommended as a minimum volume of 50 PAH or CTEPH cases managed per year^1,2^. Considering the highly specialized nature of CTEPH care, additional considerations in assessing clinical competence should be considered^4^.

In the CHEST-1 study, exemption from the central adjudication and local operability evaluation were permitted, if more than 20 PEA operations per year were undertaken by a participating center^138^. The bulk of the adjudication of operability was rendered by the central committee whose members conducted over 50 PEA operations per year. Additionally, the central adjudication committee had twice the rate of assessment of operability relative to the local adjudication committee. Conclusions taken from the international CTEPH registry indicate a trend with the best in-hospital and 1-year post-operative mortality rates from centers performing higher volumes of PEA surgery. The best results have been observed from centers performing more than 50 operations per year^95^.

It is likely that this observation does not take into account the relative differences in case complexity, with potentially more challenging cases referred to higher-volume PEA centers. An increased focus has been put on the importance of surgical center experience in the UK national registry of PEA patients^101^. This report clearly shows, that the second group of 500 consecutive operations reported substantially lower in-hospital mortality rates relative to the initial group of 500 operated cases^101^. Therefore, the ability for high-volume centers to conduct more distal endarterectomy requires stratification of surgical center expertise^84^.

Similar observations apply to BPA. BPA success is closely depended on expertise. The safety and efficacy reports of the refined BPA techniques from Japan are from centers performing the highest volume of procedures (usually >100 per year)^109,125^. In conclusion, a CTEPH expert center should be able to assess and provide all established treatment methods with results that are comparable or exceeding those reported^4^.

PEA is the treatment of choice and should be offered for operable CTEPH patients. Currently, the strongest level of evidence supports the initiation of medical therapy and consideration of BPA for those deemed inoperable. Patients with persistent/recurrent symptomatic PH after PEA surgery should receive medical therapy and be considered for BPA or reoperative PEA in cases of significant re-occlusion^139^. Finally, given the subjectivity and complexity of operability assessment, patients may be initially deemed as inoperable to receive PEA surgery with or without treatments for inoperable CTEPH. Therefore, the new algorithm gives space for fluidity between these treatment modalities as information and expertise is gained.

## Conclusions

Over the past decades the research community provided major contributions to CTEPH. Countless studies yielded a better understanding of the disease itself as well as treatment strategies. Current trials will further enhance our present knowledge of the disease and move the field forward.

Following the 6^th^ World Symposium on PH, PEA surgery remains the preferred treatment of choice for operable CTEPH patients. Targeted medical therapy and BPA are promising alternative treatments options. The 6^th^ World Symposium on PH Task Force on CTEPH mandates a multimodal, individualized approach to treatment at expert centers integrating surgical, interventional, imaging and medical PH expertise with the development of clear outcomes analyses.

## Funding information and summary conflict of interest statements

MG has received compensation for scientific symposia from Actelion Pharmaceuticals Ltd., AOPOrphan Pharmaceuticals AG, Bayer Austria Ges.m.b.H., Daiichi-Sankyo Europe Ges.m.b.H., GlaxoSmithKline Pharma Ges.m.b.H., Merck Sharp & Dohme Ges.m.b.H., Servier Austria Ges.m.b.H. and United Therapeutics Corporation.
